# CURRENT APPLICATIONS OF PATIENT COMMUNICATION TECHNOLOGIES IN GERIATRIC CRITICAL CARE

**DOI:** 10.1093/geroni/igac059.1662

**Published:** 2022-12-20

**Authors:** Mary Beth Happ, Emika Miller, Judith Tate, JiWon Shin

**Affiliations:** The Ohio State University, Columbus, Ohio, United States; Ohio State University, Columbus, Ohio, United States; Ohio State University, College of Nursing, Columbus, Ohio, United States; University of California, Davis, Sacramento, California, United States

## Abstract

The availability and utility of patient-centered communication technologies in acute-critical care settings have evolved slowly over the past 30 years with wide variability, little standardization, and few randomized controlled clinical trials (RCT). The COVID-19 pandemic forced rapid expansion and use of communication technologies, particularly between patients and remote family caregivers. To capture changes responsive to the pandemic, this paper reviews current literature (< 5 years) on communication technologies in acute-critical care settings focusing on the user experience among older adult patients. We supplement these findings with case-based evidence from a pilot RCT of an electronic tablet communication application provisioned to mechanically ventilated ICU patients, and efforts toward hospital-wide implementation. Recent literature on patient communication technology consists primarily of qualitative, descriptive accounts of video communication (i.e., ICU visits) or provision of augmentative and alternative communication. Recommendations for required skills, standardization, and research regarding patient communication technology are provided.

